# Down-regulation of Toll-like receptor 4 gene expression by short interfering RNA attenuates bone cancer pain in a rat model

**DOI:** 10.1186/1744-8069-6-2

**Published:** 2010-01-20

**Authors:** Liu Si Lan, Yang Jian Ping, Wang Li Na, Jiang Miao, Qiu Qiao Cheng, Ma Zhen Ni, Liu Lei, Li Cai Fang, Ren Chun Guang, Zhou Jin, Li Wei

**Affiliations:** 1 Department of Anesthesiology, The First Affiliated Hospital of Soochow University, Suzhou, Jiangsu 215006, China; 2 Department of Anesthesiology, The Eastern Municipal Hospital of Soochow, Suzhou, Jiangsu 215001, China

## Abstract

**Background:**

This study demonstrates a critical role in CNS innate immunity of the microglial Toll-like receptor 4 (TLR4) in the induction and maintenance of behavioral hypersensitivity in a rat model of bone cancer pain with the technique of RNA interference (RNAi). We hypothesized that after intramedullary injection of Walker 256 cells (a breast cancer cell line) into the tibia, CNS neuroimmune activation and subsequent cytokine expression are triggered by the stimulation of microglial membrane-bound TLR4.

**Results:**

We assessed tactile allodynia and spontaneous pain in female Sprague-Dawley (SD) rats after intramedullary injection of Walker 256 cells into the tibia. In a complementary study, TLR4 small interfering RNA(siRNA) was administered intrathecally to bone cancer pain rats to reduce the expression of spinal TLR4. The bone cancer pain rats treated with TLR4 siRNA displayed significantly attenuated behavioral hypersensitivity and decreased expression of spinal microglial markers and proinflammatory cytokines compared with controls. Only intrathecal injection of TRL4 siRNA at post-inoculation day 4 could prevent initial development of bone cancer pain; intrathecal injection of TRL4 siRNA at post-inoculation day 9 could attenuate, but not completely block, well-established bone cancer pain.

**Conclusions:**

TLR4 might be the main mediator in the induction of bone cancer pain. Further study of this early, specific, and innate CNS/microglial response, and how it leads to sustained glial/neuronal hypersensitivity, might lead to new therapies for the prevention and treatment of bone cancer pain syndromes.

## Background

Bone metastasis-induced pain manifests as spontaneous pain, hyperalgesia, and allodynia[1]. Pain severe enough to compromise their daily lives affects 36%-50% of cancer patients[2]. To clarify the mechanisms of bone cancer pain, rat models of bone cancer pain using breast cancer cells (Walker 256 cell) have been established[3,4], and Yao et al. [4] revealed that rats with bone cancer pain were not sensitive to radiant heat pain.

Bone cancer pain appears to be mechanistically distinct compared with neuropathic or inflammatory pain states, where major differences occur in the cellular and neurochemical changes in the nervous system. There is a prominent up-regulation of glial cells in the spinal cord ipsilateral to bone cancer pain [5]. Increasing evidence indicates that glial cell activation in the spinal cord plays a critical role in the initiation and/or maintenance of pathological pain with various etiologies [6]. With regard to neuropathic pain, several neuron-to-glia activation signals have been proposed, including fractalkine acting via microglial CX3CR1, ATP acting via the microglial P2X4 receptor, or P2X7 receptor and proinflammatory cytokines (IL-1β, TNF-α, IL-6 and INF-β) release via the microglial TLR4 receptor [7-9]. TLR4 is a transmembrane receptor protein with extracellular leucine-rich repeat domains and a cytoplasmic signaling domain. In the central nervous system, TLR4 is predominantly expressed by microglia. It has been proposed that the TLR4 is the key receptor in the formation of neuropathic pain without any exogenous LPS and exogenous pathogen [10]. Tanga et al. demonstrated that sensory neuron damage leads to the release of such substances and that these stimulate microglial TLR4 in the spinal cord, initiating microglial activation [11]. Recently, Bettoni et al. reported that repeated administration of a potent TLR4 antagonist (FP-1) resulted in relief of both thermal hyperalgesia and mechanical allodynia in mice with painful neuropathy [9]. Direct evidence of TLRs mediating inflammatory pain is still lacking. Data has accumulated on TLR expression or effects of TLR ligands on microglial activation during inflammatory pain. In a rat model of complete Freund's adjuvant-induced chronic pain, increased microglial activation, accompanied by upregulation of TLR4 mRNA expression and release of TNFa, IL-1β, and IL-6, has been reported [12]. Such findings implicate participation of TLRs in inflammatory pain.

OX42, a microglial cells-specific cellular protein found in the supporting glial cells of the spinal cord, increased markedly in bone cancer pain [13]. TLRs are also expressed on the tumor cell surface, and the incidence of lung cancer in TLR4 mutant mice is 60% more than in normal mice [14]. TRL4 also mediates the destruction of first molar [15] and cranial bones [16]. These data indicate that TLR4 is closely related to the occurrence of tumors and bone destruction. Thus, we speculated that the immune response mediated by the TLR4 signaling pathway might be involved in the induction and maintenance of bone cancer pain. Blocking the TLR4 signaling pathway could potentiate analgesic effects in bone cancer pain.

RNA interference (RNAi), an accurate and potent gene-silencing method, has demonstrated the clinical potential of synthetic small interfering RNAs (siRNAs) or short hairpin RNAs (shRNAs) in dental diseases, eye diseases, cancer, metabolic diseases, and neurodegenerative disorders [17]. RNAi can selectively silence genes, and is more efficient than traditional antisense approaches [18]. The purpose of the present studies is three-fold. First, we assessed glial activation, TLR4 and cytokine expression, and behavioral hypersensitivity after Walker 256 cells inoculation in female S-D rats. Second, we ensured the in vitro effectiveness of the siRNAs in knocking down TLR4 mRNA. Third, we tested whether TLR4 blockade by intrathecal injection of siRNA could reverse well-established bone cancer pain, and not simply prevent its initial development, because reversal is the more clinically relevant endpoint. We established a role for TLR4 and CNS innate neuroimmune activation in the development of behavioral hypersensitivity in a rat model of bone cancer pain.

## Results

### Bone cancer pain caused by Walker 256 cells inoculation

**Figure 1:**
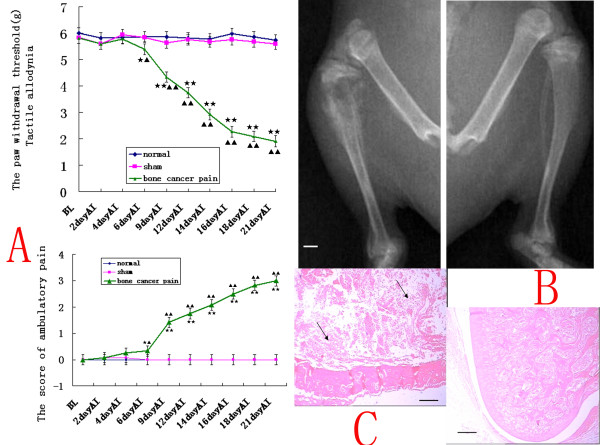
**A rat model of bone pain from metastatic bone cancer**. (A) Rats with tibia tumors after Walker 256 cells inoculation displayed both tactile allodynia and spontaneous pain. The PWL progressively decreased and the ambulatory score progressively increased on days 6 (n = 12, ANOVA_2w_, P < 0.05 pos-hoc Bonferroni, df = 2, F = 1.07), 9, 12, 14, 16, 18, and 21 (ANOVA_2w_, P < 0.01, df = 2, F = 1.13) after inoculation (AI) in the tumor-bearing group compared with sham-injected controls and normal rats. Results are given as means ± S.D, *p < 0.05 **P < 0.01 vs. normal group; ^▲^p < 0.05 ^▲ ▲^p < 0.01 vs. sham group. (B) Radiographs of the left tibiae 18 days after inoculation with Walker 256 cells and the right normal tibiae. There was bilateral cortical bone damage and large bone defects at the 18 days' tumor-bearing proximal epiphysis of the tibiae.(C) HE staining of normal (upper panel) and 18 days of tumor-bearing (lower) proximal epiphysis of the tibiae. Note that tumor cells were densely packed in the marrow cavity and induced the destruction of trabeculae. Arrows indicate tumor cells. Bar = 1 mm in B and 40 μm in C.

Rats with tibia tumors after Walker 256 cells inoculation displayed both tactile allodynia and spontaneous pain (Fig. 1A). Before Walker 256 cells inoculation, there were no significant differences in overall mean baseline paw withdrawal latency (PWL) to tactile allodynia and ambulatory score(AS) to spontaneous pain among the normal group, sham group, and bone cancer pain group(n = 12, ANOVA_2w_, PWL, P = 0.13 and AS, P = 0.39). Initially, ipsilateral tactile allodynia was observed in the sham and cancer pain groups after inoculation, but by two to three days post-surgery, thresholds in both groups returned to the baseline. In contrast, six days after inoculation, marked decreases in the PWL to tactile allodynia were observed in more than 90% of the tumor-bearing rats compared with sham-injected controls (ANOVA_2w_, P < 0.05, pos-hoc Bonferroni); at the same time, the ambulatory score began to rise (ANOVA_2w_, P < 0.05, pos-hoc Bonferroni). Subsequently, the PWL progressively decreased and the ambulatory score progressively increased in the tumor-bearing group(ANOVA_rm_, P < 0.01, pos-hoc Bonferroni).

Radiography of the left tibia showed 18 days' tumor-bearing proximal epiphysis of the tibiae, there was bilateral cortical bone damage and large bone defects (Fig. 1B). Histological studies showed that the bone marrow cavity was full of tumor cells and that the cortical bone was destroyed (Fig. 1C).

### TLR4 expression increases in the bone cancer pain model

**Figure 2:**
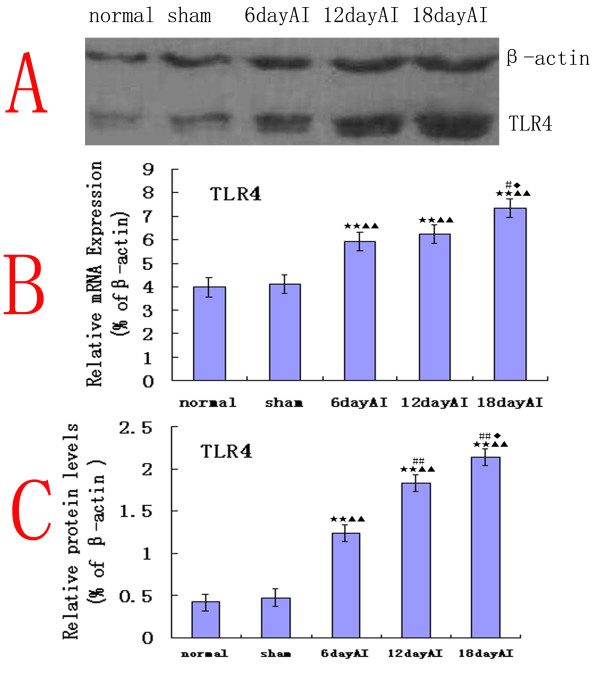
**TLR4 expression increases in the bone cancer pain model**. (B) Real-time quantitative RT-PCR analyses of mRNA temporal expression of TLR4 in rat lumbar spinal cord. The relative levels of spinal TLR4 mRNA significantly increased on days 6, 12 and18 (n = 4, ANOVA_1w_, P < 0.01, post hoc Dunnett testing, df = 3, F = 1.17) after inoculation (AI) in bone cancer pain rats compared with sham-injected controls and normal rats. The level of gene expression was calculated after normalizing against β-actin in each sample and is presented as relative mRNA expression units. (A, C) The protein level of TLR4 detected by western blotting analysis in spinal cord. A significant up-regulation of TLR4 protein level (n = 4, ANOVA_1w_, P < 0.01, post hoc Dunnett testing, df = 3, F = 1.06) on the sixth day AI. The protein levels further increased at 12 and 18 days (P < 0.01) after inoculation compared with sham-injected controls and normal rats, respectively. Each data point represents at least three independent experiments. Values are presented as mean ± SEM. *P < 0.05, **P < 0.01 vs. normal group; ^▲^P < 0.05, ^▲▲^P < 0.01 vs. sham group; ^#^P < 0.05, ^##^P < 0.01 vs. 6 day AI; ^◆^P < 0.05, ^◆◆^P < 0.01 vs. 12 day AI.

The relative levels of spinal TLR4 mRNA were significantly higher (n = 4, ANOVA_1w_, P < 0.01, post hoc Dunnett testing) in bone cancer pain rats than in sham rats, on the sixth day after inoculation (Fig. 2B). This suggests that expression of TLR4 mRNA was up-regulated by the induction of bone cancer. At the protein level, data from several western blot experiments revealed a significant up-regulation of TLR4(n = 4, ANOVA_1w_, P < 0.01, post hoc Dunnett testing) on the sixth day after inoculation (Fig. 2A, C). The protein levels further increased at 12 and 18 days after inoculation compared with sham-injected controls and normal rats, respectively. These data suggest that TLR4 expression was significantly up-regulated in the bone cancer pain model caused by Walker 256 cells inoculation.

### Microglial activation and proinflammatory cytokine expression increases in the bone cancer pain model

**Figure 3:**
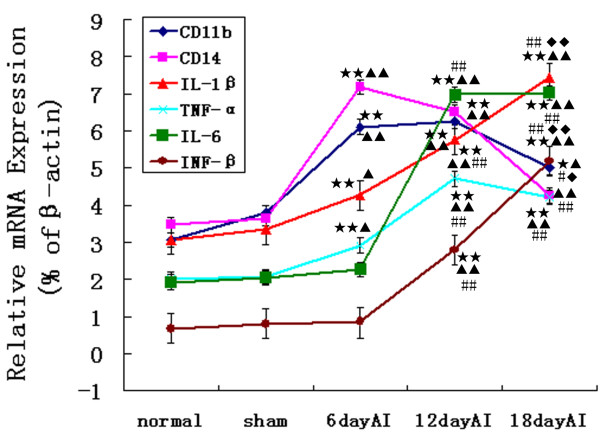
**Microglial activation and proinflammatory cytokine expression increases in the bone cancer pain model**. Real-time quantitative RT-PCR analyses of the temporal expression of microglial activation markers (CD11b, CD14) and proinflammatory cytokines (IL-6, IL-1β, TNF-α, and INF-β) mRNA in rat lumbar spinal cord. The mRNA expression levels of CD11b and CD14 significantly increased in the bone cancer pain group at six days AI and 12 days AI (n = 4, ANOVA_1w_, P < 0.01, post hoc Dunnett testing, df = 3, F = 0.98). CD11b mRNA expression was also higher at 18 days AI (P < 0.05). The bone cancer pain group also showed significant up-regulation IL-6, IL-1β, TNF-α, and INF-β mRNA relative to the normal and sham group(n = 4, ANOVA_1w_, P < 0.01, post hoc Dunnett testing, df = 3, F = 1.11), starting at day six after inoculation for IL-1β and TNF-α (P < 0.05), and at day 12 after inoculation for IL-6 and INF-β (P < 0.01). Values are presented as mean ± SEM. *P < 0.05, **P < 0.01 vs. normal group; ^▲ ^P < 0.05, ^▲▲ ^P < 0.01 vs. sham group; ^#^P < 0.05, ^##^P < 0.01 vs. 6 days AI; ^◆^P < 0.05, ^◆◆^P < 0.01 vs. 12 days AI.

A two- to three-fold increase in the mRNA expression levels of microglial activation markers CD11b and CD14 was observed in bone cancer pain rats at days 6 and 12 after inoculation (n = 4, ANOVA_1w_, P < 0.01, post hoc Dunnett testing). CD11b mRNA expression was also higher at day 18 after inoculation relative to the normal and sham group (ANOVA_1w_, P < 0.05, post hoc Dunnett testing). The bone cancer pain group also showed significant up-regulation proinflammatory cytokines mRNA (IL-6, IL-1β, TNF-α, and INF-β) (n = 4, ANOVA_1w_, P < 0.01, post hoc Dunnett testing), starting at day six after inoculation for IL-1β and TNF-α (*P *< 0.05), and starting at day 12 after inoculation for IL-6 and INF-β (*P *< 0.01) (Fig. 3).

### Identification of optimal siRNAs for knockdown of TLR4 in cell culture

**Figure 4:**
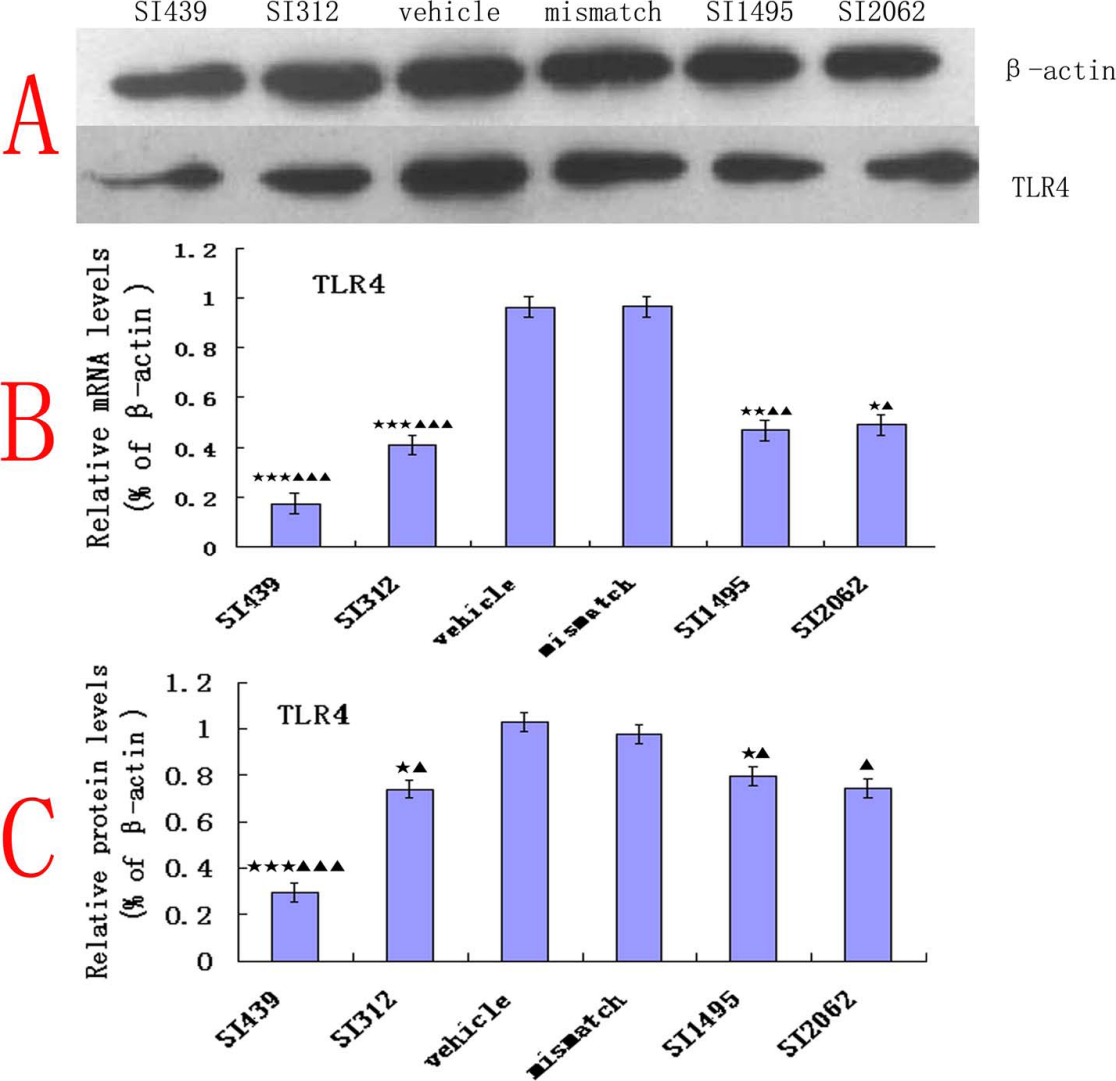
**Molecular down-regulation of TLR4 mRNA and protein upon treatment with siRNA**. (B) TLR4 mRNA levels in microglial cells line (HAPI), normalized to β-actin, were significantly reduced in TLR4 siRNA (SITLR4) versus mismatch siRNA- and vehicle-treated cells 24 h after transfection. siRNA439 was the most effective at knocking down TLR4 expression(n = 4, ANOVA_1w_, P < 0.001, post hoc Dunnett testing, df = 3, F = 1.08). (A) The TLR4 protein levels detected by western blotting analysis in HAPI 48 h after transfection, normalized to β-actin. (C) TRL4 protein levels were significantly reduced following treatment with TLR4 siRNA (SITLR4) compared with vehicle or mismatch siRNA treatment. Each data point represents at least three independent experiments. Values are presented as mean ± SEM. *P < 0.05, **P < 0.01, ***P < 0.001 vs. vehicle-treated cells; ^▲^P < 0.05, ^▲▲^P < 0.01, ^▲▲^^▲^P < 0.001 vs. mismatch siRNA-treated group.

To assure the effectiveness of the siRNA sequences for intrathecal injection, the ability of these siRNAs to knockdown TLR4 mRNA was determined *in vitro*. The TLR4 siRNA pool (2 μg) reduced TLR4 mRNA by 83% (siRNA_439_), 61% (siRNA_312_), 55% (siRNA_1495_), and 53% (siRNA_2062_), respectively, compared with a vehicle-treated group, 24 h after transfection in a microglial cells line (HAPI). This effect was not seen with scrambled duplexes (Fig. 4B). In addition to measuring the effect of the siRNA treatment on TLR4 mRNA, we also determined their effect TLR4 protein expression 48 h after transfection, by western blot analysis. TLR4 protein expression was significantly reduced following treatment with TLR4 siRNA compared with mismatched siRNA treatment (Fig. 4A,C). The most effective siRNA for knocking down TLR4 expression was siRNA_439_(n = 4, ANOVA_1w_, P < 0.001, post hoc Dunnett testing).

### Intrathecal TLR4 siRNA_439 _attenuates bone cancer pain in the rat model

**Figure 5:**
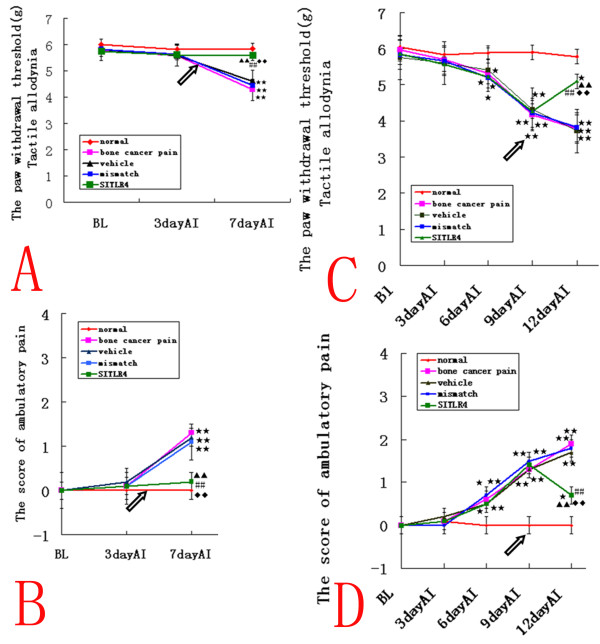
**Intrathecal TLR4 siRNA_439 _attenuates bone cancer pain in the rat model**. (A, B) In the siRNA-treated IBCP group, the PWTs were significantly higher and the ambulatory scores were significantly lower than those observed in mismatch siRNA- and vehicle-treated rats(n = 10, ANOVA_2w_, P < 0.01, pos-hoc Bonferroni, df = 4, F = 1.02), and there were no significant differences compared to normal rats. This indicated that intrathecal injection of TLR4 siRNA_439 _(SITLR4) could prevent the initial development of bone cancer pain. (C, D) In the siRNA-treated WBCP group, the PWTs were significantly elevated compared with the mismatch siRNA- and vehicle-treated rats(n = 10, ANOVA_2w_, P < 0.01, pos-hoc Bonferroni, df = 4, F = 0.96), but still lower than in normal rats (P < 0.05) Meanwhile, spontaneous pain was attenuated compared with mismatch siRNA- and vehicle-treated rats(n = 10, ANOVA_2w_, P < 0.01, pos-hoc Bonferroni, df = 4, F = 1.05). However, the ambulatory score was still higher than in normal rats (P < 0.05), which indicated that intrathecal injection of TLR4 siRNA_439 _(SITLR4) could alleviate, but not reverse, well-established bone cancer pain. Values are presented as mean ± SEM. *P < 0.05, **P < 0.01 vs. normal group; ^▲ ^P < 0.05, ^▲ ^^▲ ^P < 0.01 vs. vehicle-treated group; ^#^P < 0.05, ^##^P < 0.01 vs. bone cancer pain group; ^◆^P < 0.05, ^◆◆^P < 0.01 vs. mismatch siRNA-treated group. Arrows indicate the siRNA injection times.

In the siRNA-treated IBCP group, the PWTs were significantly higher and the ambulatory scores were significantly lower than those observed in mismatch siRNA- and vehicle-treated rats (n = 10, ANOVA_2w_, P < 0.01, pos-hoc Bonferroni), and there were no significant differences compared to normal rats (ANOVA_2w_, PWT, P = 0.93 and AS, P = 0.3) (Fig. 5A, B). No significant difference was observed between mismatch siRNA- and vehicle-treated rats, which indicated that intrathecal injection of TLR4 siRNA_439 _could prevent the initial development of bone cancer pain. In the siRNA-treated WBCP group, the PWTs were significantly elevated compared with the mismatch siRNA- and vehicle-treated rats (n = 10, ANOVA_2w_, P < 0.01, pos-hoc Bonferroni), but still lower than in normal rats (P < 0.05) (Fig. 5C, D). Meanwhile, spontaneous pain was attenuated compared with mismatch siRNA- and vehicle-treated rats(n = 10, ANOVA_2w_, P < 0.01, pos-hoc Bonferroni). However, the ambulatory score was still higher than normal rats (P < 0.05), which indicated that intrathecal injection of TLR4 siRNA_439 _could alleviate, but not reverse, well-established bone cancer pain.

### Attenuation of TLR4 expression by siRNAs

**Figure 6:**
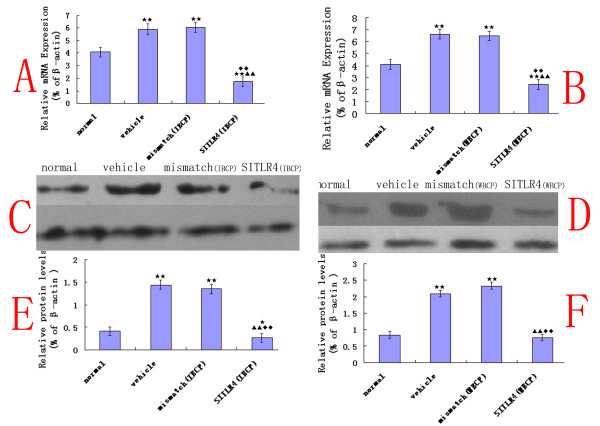
**Attenuation of TLR4 expression by Intrathecal TLR4 siRNA_439_**. (A, B) The TLR4 mRNA levels in the L4~6 lumbar spinal cord from mismatch siRNA- and vehicle- treated bone cancer pain rats were higher in IBCP group (A) and in WBCP group (B) (n = 4, ANOVA_1w_, P < 0.01, post hoc Dunnett testing, df = 3, F = 1.12)relative to that of normal rats. TLR4 siRNA_439 _(SITLR4) treatment caused a significant decrease in IBCP group, and in WBCP group in the TLR4 mRNA level compared with that in normal rats (P < 0.01). (C, D, E, F) Data from several western blotting experiments revealed a significant up-regulation of TLR4 (n = 4, ANOVA_1w_, P < 0.01, post hoc Dunnett testing, df = 3, F = 1.16) protein in mismatch siRNA-and vehicle- treated bone cancer pain rats relative to that from normal rats, whether in the IBCP group (C, E) or in the WBCP group (D, F). TLR4 siRNA_439 _treatment caused a significant decrease (n = 4, ANOVA_1w_, P < 0.01, post hoc Dunnett testing, df = 3, F = 0.96)relative the level in mismatch siRNA-and vehicle- treated bone cancer pain rats, and a significant decline compared to normal rats (P < 0.05) in the IBCP group (C, E). Values are presented as mean ± SEM. *P < 0.05, **P < 0.01 vs. normal group; ^▲ ^P < 0.05, ^▲▲ ^P < 0.01 vs. vehicle-treated group; ^◆^P < 0.05, ^◆◆^P < 0.01 vs. mismatch siRNA-treated group.

The TLR4 mRNA levels in the L4~6 lumbar spinal cord from mismatch siRNA- and vehicle- treated bone cancer pain rats were higher (62% ± 8 in IBCP group; 70% ± 6 in WBCP group, *n *= 4) relative to that of normal rats. TLR4 siRNA_439 _treatment caused a significant decrease (58% ± 8 in IBCP group (Fig. 6A), 43% ± 7 in WBCP group (Fig. 6B) (*n *= 4)) in the TLR4 mRNA level compared with that in normal rats.

At the protein level, whether in the IBCP group (Fig. 6C, E) or in the WBCP group (Fig. 6D, F), data from several western blot experiments revealed a significant up-regulation of the TLR4 (n = 4, ANOVA_1w_, P < 0.01, post hoc Dunnett testing) protein in mismatch siRNA- and vehicle-treated bone cancer pain rats relative to that from normal rats. TLR4 siRNA_439 _treatment caused a significant decrease (n = 4, ANOVA_1w_, P < 0.01, post hoc Dunnett testing) relative to the level in mismatch siRNA- and vehicle-treated bone cancer pain rats, and a significant decline compared to normal rats(P < 0.05) in the IBCP group.

### siRNA-mediated knockdown of TLR4 leads to decreased bone cancer pain-induced microglial activation and proinflammatory cytokine expression

**Figure 7:**
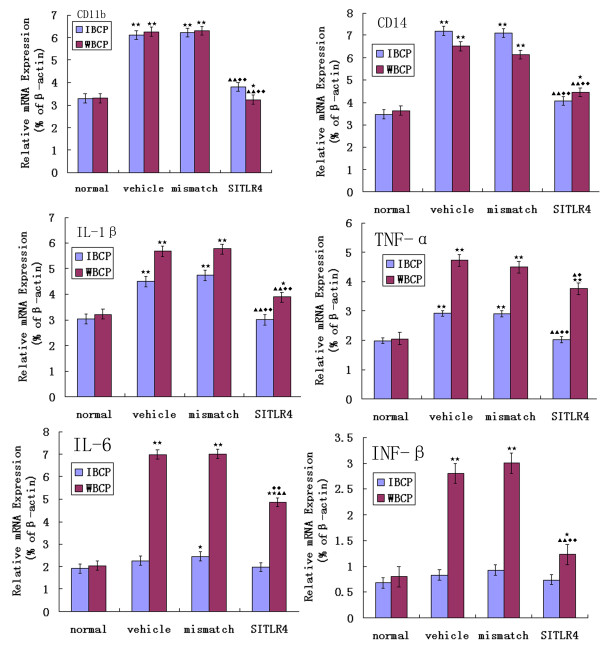
**Decreased bone cancer pain-induced microglial activation and proinflammatory cytokine expression by Intrathecal TLR4 siRNA_439_**. In both IBCP group and WBCP group, Real-time quantitative RT-PCR analyses revealed a significant down-regulation of two microglial activation markers, CD14 and CD11b compared with mismatch siRNA- and vehicle-treated rats(n = 4, ANOVA_1w_, P < 0.01, post hoc Dunnett testing, df = 3, F = 1.08). Proinflammatory cytokines mRNA expression was also significantly down-regulated, i.e., IL-1β (P < 0.01), TNF-α (P < 0.05), IL-6 (P < 0.01), and INF-β (P < 0.01) in the WBCP group; IL-1β (P < 0.01), and TNF-α (P < 0.01) in the IBCP group compared with rats treated with vehicle or the mismatch siRNA. Values are presented as mean ± SEM. *P < 0.05, **P < 0.01 vs. normal group; ^▲ ^P < 0.05, ^▲▲ ^P < 0.01 vs. vehicle-treated group; ^◆^P < 0.05, ^◆◆^P < 0.01 vs. mismatch siRNA-treated group.

Decreased expression of TLR4 was coincident with decreased expression of the microglial activation markers CD11b (48% in IBCP group; 39% in WBCP group) and CD14 (43%; 30%)(n = 4, ANOVA_1w_, P < 0.01, post hoc Dunnett testing). The decrease in TLR4 also led to a significant decrease in proinflammatory cytokines, i.e., TNF-α (31%; 20%), IL-1β (35%; 33%), IL-6 (16%; 31%), and INF-β (16%; 58%) compared with rats treated with vehicle or the mismatch siRNA (Fig. 7).

## Discussion

The data presented herein demonstrate a key role for microglial TLR4 in the induction and maintenance of behavioral hypersensitivity in rodent models of bone cancer pain. Our data also shows that transcription of the microglial activation markers, CD11b and CD14, was significantly elevated at the initiation phase of behavioral hypersensitivity, and moderately increased at the maintenance phase. These results suggest that microglia are involved in the induction and maintenance of behavioral hypersensitivity in rodent models of bone cancer pain, and are not just involved in the initiating phase, as in neuropathic pain and inflammatory pain [19,20].

The present study successfully established a female rat model of bone pain from metastatic bone cancer. What is in some ways unique about bone cancer pain is that the inflammation, tumor-released products, and tumor-induced injury to primary afferent neurons can simultaneously drive the chronic pain state [21]. There is a prominent up-regulation of glial cells in the spinal cord, ipsilateral to bone cancer pain, and a growing body of evidence suggests that glial cells in the spinal cord play an important role in pain facilitation [22]. A recent report shows that a proliferative burst of glial cells occurs in the ipsilateral dorsal horn following peripheral nerve injury, and that the majority of dividing cells were microglia [23]. The time course of microglia proliferation closely correlated with the development of neuropathic pain, suggesting an important link between microglia activation and pathogenesis of pain hypersensitivity. Although the signals that induce microglial proliferation in response to nerve injury remain incompletely clarified, it has been recently reported that, among the different receptors expressed by microglia, the TLRs, specifically TLR2 and TLR4, are a likely route for microglia activation after nerve injury and play a pivotal role in driving pain hypersensitivity[9]. Our data showed that the spinal microglial activation markers mRNA, TLR4 mRNA and TLR4 protein expression were significantly elevated in the rat model of bone cancer pain. Meanwhile, a report revealed that TLR4-deficient mice have reduced bone destruction following mixed anaerobic infection [15], which suggests that TLR4 is involved in bone cancer-induced pain perception. In a model of neuropathic pain, spinal microglia TLR4 mRNA expression is also increased after L5 nerve transection in rats [24]. TLR4-knockout and point mutant mice developed less neuropathic pain, showed reduced glial activation, and strongly decreased expression of pain related cytokines [24]. These data shed light on the mechanistic link between microglial TLR4 activation and behavioral hypersensitivity.

This is further evidenced by the present study's finding that functional knockdown of the TLR4 gene by RNAi significantly inhibited bone cancer-induced behavioral hypersensitivity. Consistent with the behavioral test, the real-time RT-PCR study and western-blot analysis demonstrated that TLR4 significantly suppresses spinal TLR4 mRNA and protein expression during bone cancer pain. These data indicate that TLR4 is involved in the spinal transmission and processing of noxious inputs from the peripheral cancer area and facilitates bone cancer hyperalgesia. In the model of neuropathic pain, TLR4 antisense oligonucleotide treatment can significantly attenuate the behavioral hypersensitivity [24]. Repeated administration of a potent TLR4 antagonist (FP-1) could also relieve thermal hyperalgesia and mechanical allodynia in mice with painful neuropathy [9]. These data indicate that TLR4 is not only involved in neuropathic pain, but also in bone cancer pain.

It should also be noted that our research shows that functional knockdown of TLR4 also reduced spinal microglial activation, and reduced the expression of mRNA for spinal proinflammatory cytokines. A previous study demonstrated that IL-1β was upregulated in a bone cancer pain rat model and that intrathecal IL-1ra produced an anti-pain effect in such a model [13,25]. The CNS innate immune response includes rapid activation of immune effectors cells and the release of proinflammatory cytokines, such as TNF-α, IL-1 β, IL-6, and IFN-β, through the activation of TLR4-MyD88-dependent or -independent pathways [26]. Furthermore, the expression of proinflammatory cytokines by activated glia after the injection cancer cells into the tibia has been shown to be a major factor contributing to the establishment of behavioral hypersensitivity [27]. As shown in the present study, the knockdown of TLR4 expression leads to attenuation of behavioral hypersensitivity, decreased glial activation, and decreased expression of proinflammatory cytokines. Thus, it is very important that TLR4 provides a mechanistic link between microglial activation, innate immunity, and the initiation of behavioral hypersensitivity in the rat model of bone cancer pain.

Like many chronic pain states, bone cancer pain becomes more severe with disease progression, requiring higher doses of analgesics to control the pain[28]. In the present report, we show that intrathecal TLR4 siRNA could prevent bone cancer-induced tactile allodynia and spontaneous pain at an early stage of tumor growth. However, at the late stage, intrathecal TLR4 siRNA can only attenuate, but not completely block, well-established bone cancer pain. This finding suggests that TLR4 is the main mediator in the induction of bone cancer pain, and that there is a potential role for other receptors to be involved in maintaining the pain state [29,30]. These might include bradykinin, P2X3, TRPV1, and prostaglandin receptors, acid-sensing ion channel 3 and voltage-gated sodium channels, and unique glial/neuronal signals like fractalkine. Thus, our results underscore the complexity of CNS cascades and mediators that may underlie neuronal sensitization, the pathological manifestation of cancer pain.

In vivo delivery of siRNAs into the central nervous system is complicated by the fact that oligonucleotides do not efficiently cross the blood-brain barrier. Recently, chemical modifications of siRNAs have become essential for achieving high levels of gene silencing, enhancing plasma stability, and increasing in vivo potency, combined with a low degree of undesired effects [31]. The present study applied RNAi technology that was improved in two ways: (i) chemical modification of the siRNA. The introduction of certain constituents, such as fluorine, into the ribose 2' position of the primers renders siRNAs more resistant to nuclear acid enzymes. In addition, the 3'-end of the siRNA antisense strand has the special role of identification of the target mRNA, and chemical modification at the 3'-end of the antisense strand will lead to a significant increase in interference. Therefore, we modified our siRNAs with one 2'-FU substitution at the 3'-end of the sense strand, but left the antisense chain unmodified, by which we hoped for enhanced nuclease resistance combined with good specificity. (ii) Application of an appropriate delivery system. We applied in vivo jetPEI™ (polyethyleneimine, PEI) as the siRNA delivery system. PEI is a cationic polymer nanoparticle that, compared with liposomes or viral vector, can decrease cytotoxicity, and can avoid potential vector immunogenicity and tumorigenicity, when used as an siRNA delivery system. Growing evidence indicates that PEI-siRNA is an ideal tool for inhibiting specific gene expression [31].

## Conclusion

Our results provide evidence for a role of the key innate immune receptor, TLR4, in a rat model of bone cancer pain. The ability of TLR4 to activate a pathway leading to central sensitization and behavioral hypersensitivity could provide an opportunity for regulating glial activation, and thus, alleviating chronic pain, such as bone cancer pain.

## Experimental procedures

### Animals

Sprague-Dawley rats (150-180 g) provided by the Experimental Animal Center of Soochow University, were kept under a 12 h/12 h light-dark cycle regime, with free access to food and water. All surgical and experimental procedures were reviewed and approved by the Animal Care and Use Committee of the Soochow University. Animal treatments were performed according to the Guidelines of the International Association for the Study of Pain (Zimmermann, 1983).

### Intrathecal Catheterization

Five days before intra-tibial injection of Walker256 cells, under anesthesia with pentobarbital sodium (40 mg/kg, i.p.), a polyurethane intrathecal catheter with an inner diameter of 0.3 mm and an outer diameter of 0.6 mm (R-ITC, Pittsburgh, PA) was inserted 5 mm cephalad into the rat lumbar subarachnoid space at the L4-L5 intervertebrae. The tip of the catheter was located near the lumbar enlargement of the spinal cord, to administer the drugs intrathecally, according to the modification of a method described previously [32]. The catheter was tunneled subcutaneously and externalized through the skin in the neck region. The volume of dead space of the intrathecal catheter was 10 μl. To avoid occlusion of the catheter, 10 μl of normal saline was injected via a catheter on alternate days until the end of the experiment. Three days later, 2% lidocaine (10 μL) was injected intrathecally to rats that showed no impaired movement, and animals that showed lower limb paralysis within 30 s indicated successful catheterization.

### Bone cancer model

Walker 256 cells injection protocol was performed as described previously [33]. In brief, under anesthesia with pentobarbital sodium (40 mg/kg, i.p.), rats were fixed, and the left tibia was prepared for surgery. A skin incision was made parallel to the tibia to expose the tibial plateau. A needle was then inserted into the medullary canal to create a pathway for the Walker 256 cells. A depression was then made using a microinjector. Sham animals (n = 10) were generated with an injection of 5 μl of saline into the intramedullary space of the tibia, whereas Walker 256-injected animals (n = 10) were injected with media containing 10^5 ^Walker 256 cells in 5 μl. For all animals, the injection site was sealed with a medical glue to confine the cells within the intramedullary canal, followed by irrigation with sterile water (hypotonic solution). Finally, incision closure was achieved using the medical glue.

### Behavioral studies

#### Tactile allodynia

Tactile allodynia was assessed by using von Frey filaments (Stoelting, Wood Dale, IL). Animals were placed in individual plastic boxes (20 cm × 25 cm × 15 cm) on a metal mesh floor and allowed to acclimatize for 30 min. The filaments were presented, in ascending order of strength, perpendicular to the plantar surface with sufficient force to cause slight bending against the paw and held for 6-8 s. Brisk withdrawal or paw flinching were considered as positive responses. The paw withdrawal threshold (PWT) was determined by sequentially increasing and decreasing the stimulus strength (the "up-and-down" method), and the data were analyzed using the nonparametric method of Dixon, as described by Chaplan et al. [34].

#### Spontaneous pain

Spontaneous pain was evaluated by ambulatory score in the tumor-injected hind limb. The scoring of each animal was characterized as follows: normal activities (0); mild claudication (1); moderate claudication (2); severe claudication (3); and disuse (4). All of the behavioral data were obtained using at least twelve animals for each time point or group.

#### Assessing the extent of bone destruction

##### Radiology

To confirm cancer development in the tibia, rats were X-rayed six, 12, and 18 days after surgery. Anaesthetized rat hind limbs were placed on X-ray film (Kodak, Ita1v) and exposed to an X-ray source (Emerald 125) for 1/20 s at 40 KVP. The radiographic images were visually quantified using the scale developed by Schwei et al [35].

##### Histochemical staining

After demineralizing in EDTA (10%) for 2-3 weeks, the tibiae were embedded in paraffin, and 10 μm sections were cut and stained with Harris' hematoxylin and eosin (HE) to verify cancer cell infiltration and bone destruction.

##### Small interfering RNA (siRNA) constructs

Four different siRNA duplexes targeting the S-D rat TLR4 were selected using standard siRNA design rules. Additionally, a scrambled sequence was designed as a mismatch control. BLAST search of nucleotide sequences in the GenBank database showed no substantial homology with other genes. The sequences of these five siRNA constructs are shown in Table 1. All siRNA duplexes were chemically synthesized by Genepharma (ShangHai, China). The siRNA stocks were aliquoted and stored at -80°C, at a concentration of 200 μM in annealing buffer as previously described [36].

**Table 1:** SiRNA oligo

Gene	Primer forward	Primer reverse
MC	UUCUCCGAACGUGUCACGUTT	ACGUGACACGUUCGGAGAATT

312	CCUUGGUACUGACAGGAAATT	UUUCCUGUCAGUACCAAGGTT

439	GCUUAUAUCCUUAAAGAAATT	UUUCUUUAAGGAUAUAAGCTG

1495	GGAUCUUUCUAAAUGCCAATT	UUGGCAUUUAGAAAGAUCCAG

2062	CGAGCUGGUAAAGAAUUUATT	UAAAUUCUUUACCAGCUCGTT

Sequences of short interfering RNA (siRNA) constructs used in this study. In addition to a mismatch control siRNA construct, foure different siRNA duplexes were designed that targeted the coding region of the rat TLR4 mRNA (NM_019178).

##### In vitro analysis of siRNA-mediated knock down of TLR4 inHAPI cells

Microglial cells (HAPI cell line; a gift from Dr. Si JH, NanTong University) were maintained in Dulbecco's modified Eagle's medium (DMEM) supplemented with 10% fetal bovine serum, penicillin (100 U/ml), and streptomycin (100 μg/ml). One day before transfection, the cells were trypsinized and resuspended in DMEM without antibiotics, and plated into a 24-well plate at a density of 1.5 × 10^5 ^cells per 0.6 ml per well. Cells were transfected with 2 μg of siRNA/Lipofectamine™ 2000 (Invitrogen, Carlsbad, CA) complexes in 1:3 ratio (w/v) per well (four gene-specific siRNAs or a mismatch control siRNA). Forty-eight hours after transfection, the cells were harvested for western blot analysis of the level of knockdown proteins by the siRNAs. For targeting and detection of the mRNA levels, after 24 hr of transfection, RNA was isolated from the cells using RNAqueous™ -96 (Ambion), and subjected to Quantitative RT-PCR.

#### siRNA delivery

Four days after cancer cells injection, rats showing general good health with no signs of distress and no marked weight loss were selected for RNA delivery. The rats were divided into siRNA, mismatch RNA, and vehicle groups, with at least six rats per group. To increase the intracellular stability of the siRNAs, siRNAs for vivo administration were modified with one stabilizing 2'-FU substitution at the 3'-end of the sense strand. siRNA or mismatch RNA complexes were prepared immediately prior to administration by mixing the RNA solution (200 μM in annealing buffer) with a transfection reagent, in vivo jetPEI™ (Polyplus, French), in a ratio of 1:4 (w:v). At this ratio, the final concentration of RNA as an RNA/lipid complex was 2 μg in 10 μl. siRNA or mismatch RNA, or in vivo jetPEI™ alone (defined as vehicle) in 10 μl was delivered to the lumbar region of the spinal cord via the i.th. catheters. The catheters were flushed with 10 μl of sterile saline. Injections were given daily for three consecutive days. Nociceptive testing and tissue harvesting were carried out 24 hr after the last injection.

#### Real-time RT-polymerase chain reaction (RT-PCR)

HAPI Cells were rinsed twice with PBS and treated with the RNA extraction reagent Trizol (Invitrogen); Lumbar spinal cords (L4 and L5), taken from the rats and preserved in RNA *later *(Ambion), were homogenized using a Tissuelyser (MM300, Qiagen) with a single 6.5 mm stainless steel ball per sample. Total RNA was isolated from the homogenate using an RNeasy Plus Mini Kit according to the manufacturer's recommended protocol. Total RNA was precipitated by centrifugation at 20,000 × g for 15 min at 4°C, washed twice with ethanol, and quantified by UV spectrophotometry at 260 nm. Only RNA with a 260/280 ratio >2.0 was used for the reverse transcription (RT) reaction. TaqMan primers and probes were designed with PrimerExpress software (ABI). The sequences of the rat primers and probe for TLR4 (GenBank accession # NM_019178), two microglial activation markers: CD11b (NM_012711) and CD14 (NM_021744), and cytokine:IL-6 (NM_012589), IL-1β (NM_031512.2), TNF-a (NM_012675.2), and INF-β (NM_019127.1) are shown in Table 2.

**Table 2:** Primer and TaqMan probe sequences for the rat genes characterized in this experiment

Gene	Primer forward	Primer reverse
β-actin	5'-CACCCGCGAGTACAACCTTC-3'	5'-CCCATACCCACCATCACA CC-3'
Probe*	5'-FAM-TCCTACCCCCAATGTGTCCGTCGTG-TAMRA-3'	

TLR4	5'-AGCTTTGGTCAGTTGGCTCT-3'	5'-CAGGATGACACCATTGAAGC-3'
Probe*	5'-FAM-AACAGCAACCTCTAAAGCTCATGGCA-TAMRA-3'	

IL-6	5'-AAGGACCAAGACCATCCAAC-3'	5'-ACCACAGTGAGGAATGTCCA-3'
Probe*	5'-FAM-TCGGCAAACCTAGTGTGCTATGCC-TAMRA-3'	

IL-1β	5'-GCAACTGTCCCTGAACTCAA-3'	5'-TGTCAGCCTCAAAGAACAGG-3'
Probe*	5'-FAM-TCATTCTCCTCACTGTCGAAAGCTGC-TAMRA-3'	

TNF-a	5'-CTAACTCCCAGAAAAGCAAGCAA-3'	5'-CCTCGGGCCAGTGTATGAGA-3'
Probe*	5'-FAM-CAGCCAGGCAGGTTCCGTCC-TAMRA-3'	

INF-β	5'-CAGCTGAATGGAAGGCTCAAC-3'	5'-CGGGTGCATCACCTCCATA-3'
Probe*	5'-FAM-CAGCTACAGGACGGACTTCAAGATC-TAMRA-3'	

CD11b	5'-GCCTCCAAGTCCGCAAGAA-3'	5'-TCATAAGTGACAGTGCTCTGGATGT-3'
Probe*	5'-FAM-ACCAAGGACAGGCTGCGAGA AGGA-TAMRA-3'	

CD14	5'-CGGGAACTGACTCTTGAAAACC-3'	5'-CATCCAGAAGCGGCGAAA-3'
Probe*	5'-FAM-CGAGGTAACCGGCACCGCG-TAMRA-3'	

*The TaqMan probe has a reporter fluorescent dye, 6-carboxyfluorescein (FAM), at the 5' end, and a fluorescence dye quencher, 6-carboxytetramethylrhodamine (TAMRA), at the 3' end.

Quantitative PCR was carried out using MJ Research PTC-100 with TaKaRa Ex Taq R-PCR Version 2.1 (TaKaRa, Dalian, China) and Evagreen™ (Biotium, Inc., CA, United States) according to manufacturer's instructions. The PCR reactions were prepared using the components from the Platinum qRT-PCR kit and assembled according to the manufacturer's instructions (Qiagen). The final concentrations of the primers and probe in the PCR reactions were 200 nM and 100 nM, respectively. The fluorogenic probes were labeled with 6-carboxyfluorescein (6FAM) as the reporter and 6-carboxy-4,7,2,7'-tetramethylrhodamine (TAMRA) as a quencher. Each 10 μl PCR reaction contained 2 μl (10 ng) of total RNA. Six replicates of each RT-PCR reaction were performed in a 384-well plate according to the following protocol: 1 cycle for 10 min at 50°C, followed by a 15-min cycle at 95°C, followed by 45 cycles at 95°C for 15 s and 60°C for 1 min. A eukaryotic 18S rRNA endogenous control probe/primer set (ABI) was used as an internal control for RNA quality.

#### Protein extraction and western blot analysis

HAPI Cells (rinsed twice with PBS), Lumbar spinal cords (L4 and L5) taken from the rats, cells, and tissue samples were homogenized in lysis buffer (pH 7.9: HEPES 10 mmol/L, Na_3_VO_4 _1 mmol/L, MgCl_2 _1.5 mmol/L, KCl 10 mmol/L, NaF 50 mmol/L, ethylenediaminetetraacetic acid (EDTA) 0.1 mmol/L, Ethylene glycol-bis(beta-aminoethyl ether)-N, N, N', N'-tetra acetic acid (EGTA) 0.1 mmol/L, phenylmethylsulfonyl fluoride (PMSF) 0.5 mmol/L, dithiothreitol (DTT) 1 mmol/L and 0.02% protease inhibitor cocktail). The homogenates were incubated for 30 min in ice-cold water with constant agitation and then centrifuged at 13 000 g for 15 min at 4°C. The supernatants were used for Western blot analysis. Protein concentrations were determined using the Bradford method [37] and the protein samples were stored at -80°C.

Protein samples were dissolved in 4 × sample buffer (Tris-HCl 250 mmol/L, Sucrose 200 mmol/L, DTT 300 mmol/L, 0.01% Coomassie brilliant blue-G, and 8% sodium dodecyl sulfate, pH 6.8), and denatured at 95°C for 5 min. Equivalent amounts of protein (40 μg) were separated using 10% SDS-polyacrylamide gel electrophoresis (PAGE) and transferred onto a nitrocellulose membrane. Membranes were blocked with 5% fat-free milk solution overnight at 4°C. Samples were probed with 1:200 dilution of goat polyclonal antibodies against TLR4 (Santa-Cruz Biotechnology, Inc., Santa Cruz) and a 1:5000 dilution of rabbit anti- goat HRP-conjugated IgG (Huamei Chemical Corp., China). A rat monoclonal antibody against beta-actin (Sigma) was used as a loading control. Bands were revealed using an ECL kit (Pu fei Chemical Corp., Shanghai, China). Scanning densitometry was used for semiquantitative analysis of the data.

### Statistical analysis

All data are presented as mean ± standard error of mean(S.E.M.). Data from the western-blot and RT-PCR studies were accomplished using a one-way analysis of variance (ANOVA_1w_) followed by post hoc Dunnett testing. Data from the nociceptive tests were analyzed using a two-way ANOVA (ANOVA_2w_) to analyze differences between groups at different time points, while repeated-measures ANOVA (ANOVA_rm_) was used to evaluate efficiency of treatment along different time points within groups; pos-hoc Bonferroni's test was used to detect significant differences for both ANOVA analysis. A value of p < 0.05 was considered a significant difference.

## Competing interests

The authors declare that they have no competing interests.

## Authors' contributions

LSL, LL, LCF, RCG, LW, and LJ carried out the animal surgery and behavior testing; LSL, RCG, LW, and LJ carried out the RNAi experiments in vivo; LSL, YJP and WLN carried out the data analysis, wrote the manuscript, and interpreted the data. LSL, LM, and MZN participated in the RNAi experiments in vitro. LSL, QQC, and MZN carried out RT-PCR experiments and western blot analysis; YJP conceived the study, and participated in its design and coordination. All authors have read and approved the final manuscript.
